# Spontaneous and deliberate creative cognition during and after psilocybin exposure

**DOI:** 10.1038/s41398-021-01335-5

**Published:** 2021-04-08

**Authors:** N. L. Mason, K. P. C. Kuypers, J. T. Reckweg, F. Müller, D. H. Y. Tse, B. Da Rios, S. W. Toennes, P. Stiers, A. Feilding, J. G. Ramaekers

**Affiliations:** 1 Department of Neuropsychology and Psychopharmacology, Faculty of Psychology and Neuroscience, Maastricht University, P.O. Box 616, 6200 MD Maastricht, the Netherlands; 2 Department of Psychiatry (UPK), University of Basel, Basel, Switzerland; 3 Institute of Legal Medicine, University of Frankfurt, Kennedyallee 104, D-60596 Frankfurt/Main, Germany; 4 The Beckley Foundation, Beckley Park, Oxford, OX3 9SY UK

**Keywords:** Neuroscience, Human behaviour

## Abstract

Creativity is an essential cognitive ability linked to all areas of our everyday functioning. Thus, finding a way to enhance it is of broad interest. A large number of anecdotal reports suggest that the consumption of psychedelic drugs can enhance creative thinking; however, scientific evidence is lacking. Following a double-blind, placebo-controlled, parallel-group design, we demonstrated that psilocybin (0.17 mg/kg) induced a time- and construct-related differentiation of effects on creative thinking. Acutely, psilocybin increased ratings of (spontaneous) creative insights, while decreasing (deliberate) task-based creativity. Seven days after psilocybin, number of novel ideas increased. Furthermore, we utilized an ultrahigh field multimodal brain imaging approach, and found that acute and persisting effects were predicted by within- and between-network connectivity of the default mode network. Findings add some support to historical claims that psychedelics can influence aspects of the creative process, potentially indicating them as a tool to investigate creativity and subsequent underlying neural mechanisms. Trial NL6007; psilocybin as a tool for enhanced cognitive flexibility; https://www.trialregister.nl/trial/6007.

## Introduction

Creativity is an essential cognitive ability linked to all areas of our everyday life, allowing us to adapt to an ever-changing environment and come up with ways to solve problems. Although arguably difficult to define, the creative process has been viewed as a dynamic process^1,2^, requiring shifting between different modes of thought in order to reach an end result^3^. These modes include divergent thinking (DT), which consists of generating novel and original ideas, and convergent thinking (CT), the subsequent evaluation of generated ideas in regards to their usefulness and effectiveness^4,5^. Importantly, as well as being an essential process for everyday functioning, the (in)ability to think “outside of the box” has also been associated with psychological disorders, such as depression and anxiety^6,7^. Here, it has been regarded both as a transdiagnostic process, i.e., a risk or maintaining factor shared across disorders, and as a potential therapeutic intervention, promoting adaptive interpretations and coping strategies^7,8^. Thus taken together, finding a way to enhance creative thinking is of broad interest among multiple disciplines, ranging from industry to psychopathology.

Over the years, a number of anecdotal reports have accumulated suggesting that the consumption of serotonin (5-HT) 2A agonist psychedelic drugs^9^, like lysergic acid diethylamide (LSD), psilocybin, and mescaline, can enhance creativity^10,11^. Famous examples of psychedelic-affiliated creative breakthroughs span the fields of science, technology, and art; including Kary Mullis’ discovery of the polymerase chain reaction, the 1960’s California-based computer industry, and the literary works of authors, such as Aldous Huxley and Ken Kesey^10,11^. That said, although there has been much historical interest in the ability of psychedelics to enhance creative capacity, the scientific literature is largely lacking. Initial scientific studies into the effects of psychedelics on creativity date as far back as the 1950’s, and ran until the drugs were banned in the United States in the late 1960s (refs. ^10,11^). However, findings of these studies were largely inconclusive, and limited in regards to present-day methodology^12–14^. Instead, preliminary contemporary work provides stronger indications that psychedelics can affect creativity-related constructs^1,15–19^, with phenomenological reports suggesting psychedelics induce a hyper-associative state of cognition^1^, and pseudo-experimental studies finding acute^20^ and subacute^21–23^ increases in DT or CT after consumption of a psychedelic in a naturalistic setting. Overall, evidence suggests that psychedelics can mediate changes in particular constructs of creativity, however direct, experimental evidence is lacking.

Importantly, if psychedelics can mediate changes in particular dimensions of the creative process, they could be used as a potentially novel tool to investigate these processes^1^, as well as the underlying neural mechanisms^24^. In regards to creative cognition, previous research has consistently implicated the coordination of three resting-state networks (RSNs); the default mode network (DMN), suggested to support idea generation, the frontoparietal control network (FPN), supporting idea evaluation, and areas of the salience network (SN), suggested to facilitate the shift between the internally (DMN) and externally (FPN) oriented cognitive networks^25–27^. Although usually opposing neural processes, the DMN and FPN have been found to interact during creative cognition, suggesting a dynamic shift between these networks facilitates the shift between idea generation and evaluation across time^25^. Recently, this shift between neurocognitive dissociable states has been further conceptualized into a new theory (the dynamic framework of thought^1,28^), suggesting that creative thought is an alternation between states with low (DMN driven) to high (FPN driven) cognitive constraints, operating on mnemonic information from the medial temporal region. Interestingly, the authors incorporated the psychedelic state into this model^1^, suggesting that psychedelics represent a state of unconstrained cognition, with neural evidence stemming from previous imaging studies which suggest they acutely induce a flexible brain state^29–31^, characterized by an acute disintegration of normally highly organized activity within RSNs^32–36^, a simultaneous widening of dynamic repertoires of connectivity states^37^, and increased coupling of RSNs that are usually anticorrelated^34,35,38–40^. Changes in connectivity have also been found to outlast the acute stage, with reports of increased within-network DMN connectivity 1-day post-drug administration^41–43^, and increased between-network connectivity up to 1-month post-drug administration^44,45^.

Overall, previous research suggests an acute and even persisting effect of psychedelics on creativity-related cognition and neural mechanisms. However, no study has yet directly assessed the (sub)acute effect of a psychedelic on the two constructs of creativity (DT and CT) in a controlled trial, or its relationship to drug-induced brain changes. Therefore, the aim of this study was twofold: to assess the acute and persisting (7-day post-drug administration) effect of the classic psychedelic, psilocybin, on divergent and CT; and to assess (acute) biological predictors of changes in these constructs. To do this, divergent and CT were quantified via two widely used cognitive tasks, the alternate uses test (AUT)^46^ and the picture concept test (PCT)^20^. As evidence suggests a neurocognitive distinction between deliberate (task-based) creative cognition and spontaneous (insight) creative cognition^47,48^, subjective feelings of spontaneous creative thinking were also assessed, via a well-established altered states of consciousness questionnaire^49^. Then, the relationship between cognitive and subjective ratings of creativity, within- and between-network connectivity of the DMN, FPN, and SN, and glutamate concentrations in the medial prefrontal cortex (mPFC) and hippocampus, were evaluated. The latter was of interest as evidence suggests that network connectivity changes during the psychedelic state may result from changes in local network integrity due to downstream stimulation of glutamatergic signaling^43,50,51^, this is of particular interest in the mPFC and hippocampus due to their involvement in the FPN and DMN, and their recruitment in creative cognition^52–54^.

Resting-state functional magnetic resonance imaging (rsfMRI) was used to assess RSN functional connectivity (FC), and ultrahigh field (7T) proton magnetic resonance spectroscopy (MRS) was used to assess glutamate concentrations in designated brain areas. Overall it was hypothesized that psilocybin would have an acute and persisting increase on outcome measures of DT, which would be predicted by within-network FC of the DMN. It was further hypothesized that psilocybin would decrease CT acutely^20^, which would be predicted by alterations in between-network FC of the DMN and FPN.

## Materials and methods

A detailed description of the experimental procedure is provided in the Supplementary Methods and briefly summarized here.

The present study was conducted between July 2017 and June 2018 at Maastricht University, employing a balanced randomized (1:1), placebo-controlled, double-blind, parallel-group design. Sixty healthy participants, with previous experience with a psychedelic drug but not within the past 3 months, were allocated to a treatment condition (0.17 mg/kg psilocybin or placebo, p.o.). Groups were matched for age, sex, and education level. Full demographic information on the sample is available in Mason et al.^51^.

Participants visited the lab on three separate occasions. The first visit included a familiarization with testing day procedures and completion of the creativity tasks (baseline). The second visit consisted of the formal testing day, with treatment administration (psilocybin or placebo), fMRI, and completion of the creativity tasks (acute). The third testing day took place 7 days after the acute testing day, and included the final completion of the creativity tasks (follow-up).

This study was conducted according to the code of ethics on human experimentation established by the declaration of Helsinki (1964) and amended in Fortaleza (Brazil, October 2013) and in accordance with the Medical Research Involving Human Subjects Act (WMO) and was approved by the Academic Hospital and University’s Medical Ethics committee. All participants were fully informed of all procedures, possible adverse reactions, legal rights and responsibilities, expected benefits, and their right for voluntary termination without consequences. The present data were part of a larger clinical trial (Netherlands Trial Register: NTR6505) of which parts have been previously published^51^.

### Creativity tasks

Parallel (different) versions of each task were used on the baseline, acute, and follow-up testing days. All responses were scored afterward by two independent raters who were blind to the treatment condition. The interrater reliability was calculated before averaging the ratings.

#### Picture concept task

The PCT was administered during peak drug effects (~120 min post treatment), as an assessment of both convergent and DT, and in accordance with previous studies^20–23^. In brief, the PCT consists of 17 stimuli, each containing between 4 and 12 color pictures shown in a matrix. Participants were given 30 s per stimulus to find an association between one of the pictures in each row. Specifically, they were asked first to provide the correct solution, as there is only one correct answer. The number of correct answers served as the dependent measure of CT. In order to assess DT, participants were then asked to provide as many alternative answers as possible, which are used to calculate several parameters—i.e., fluency, originality, and the ratio of both. Fluency is defined as the number of alternative associations; originality is calculated by evaluating the originality of the alternative association relative to those provided by all other participants in a session; the ratio score is then calculated by originality/fluency.

#### Alternative uses task

The AUT was administered in accordance with previous publications, at ~130 min post treatment, as an assessment of DT^46^. Participants were asked to list as many possible uses for two common household items and given 3 minutes per item to do so. Dependent outcome variables were the same as with the PCT, with an addition of the dimension “novelty”. Novelty was assessed in order to separate newly generated ideas from recollection of old ideas from memory, and was done so by asking participants after idea generation to indicate if they had given any responses that were completely new to them (i.e., they had never thought of this use before, had never seen it in a movie, etc.)^55^. The number of new responses were then summed up. Additional outcome measures of the AUT that have been assessed in other studies^56^, but were out of the scope of this study, can be found in Supplementary Results.

### Subjective state

The five dimensions of altered states of consciousness (5D-ASC) scale^49,57^ was administered 360 min after drug administration, as a retrospective measure of drug effects. Of specific interest for this study was the subscale “insightfulness”, consisting of three questions conceptually related to spontaneous creative thinking: “I felt very profound”, “I had insights into connections that had previously puzzled me”, and “I had very original thoughts”.

### Image acquisition

Participants underwent structural MRI (50 min post treatment), single-voxel proton MRS in the mPFC (65 min post) and hippocampus (95 min post), and rsfMRI (102 min post), during peak subjective drug effects. Images were acquired on a MAGNETOM 7T MR scanner. At rest, 258 whole-brain EPI volumes were acquired (TR = 1400 ms; TE = 21 ms; field of view (FOV) = 198 mm; flip angle = 60°; oblique acquisition orientation; interleaved slice acquisition; 72 slices; slice thickness = 1.5 mm; voxel size = 1.5 × 1.5 × 1.5 mm). During scanning, participants were shown a black cross on a white background, and instructed to look at the cross while attempting to clear their mind, and lay as still as possible. The resting-state instructions are important to note when comparing results across studies, as different pre-scanning instructions have been found to induce distinct resting brain states during fMRI^58^.

Spectroscopic voxels were placed by a trained operator at the mPFC (voxel size = 25 × 20 × 17 mm^3^) and the right hippocampus (voxel size = 37 × 15 × 15 mm^3^). Spectra were acquired with the stimulated echo acquisition mode^59^ sequence (TE = 6.0 ms, TR = 5.0 s, 64 averages). Outcome measures for MRS were concentration ratios of glutamate to total creatine (tCr, creatine + phospho-creatine).

Detailed information regarding image acquisition and MRS quantification is previously published^51^.

### Processing of imaging data

Resting-state data were processed and analysed using the CONN toolbox 18.b (ref. ^60^). All volumes were realigned, unwarped, segmented into gray and white matter and cerebrospinal fluid, normalized into a standard stereotactic space (Montreal Neurological Institute), and smoothed with a 6 mm full-width at half-maximum Gaussian kernel. Independent component analysis (ICA) was performed using group-ICA procedures implemented in the CONN toolbox, following previously described methods^61^. Independent components were restricted to 20, in order to allow comparisons with established RSNs^62^ and previous studies on psilocybin^38^ and LSD^34,35^. Detailed information on the ICA analysis is described in Mason et al.^51^.

Spectroscopy data, to assess glutamate concentrations in the mPFC and the hippocampus, were analysed with LCModel version 6.3-1H, as described in Mason et al.^51^.

### Pharmacokinetic measures

Venous blood samples were collected at regular time points after treatment administration (80, 150, and 360 min) in order to assess blood serum concentrations of psilocin, the main metabolite of psilocybin.

### Statistics

#### Sample size calculation

This study is part of a larger trial, assessing effects of psilocybin on creativity, cognitive flexibility, brain connectivity, and glutamate concentrations. In regards to behavioral measures, our power analysis (GPower; alpha = 0.05 two-tailed; power = 0.8) for measures of creativity and cognitive flexibility indicated that the required sample size for detecting medium effects is *N* = 48. We opted to include 60 participants in the project to ensure sufficient power to be able to detect subtler effects. In regards to power analysis for imaging data, this is quite complex, with the most common approach in the neuroimaging community is to estimate the number of subjects from previous empirical experience in the field. Previous MRS and FC studies have employed 10–21 subjects for showing significant drug effects^63–65^. Based on these experiences, we expected that we would be able to demonstrate drug effects on MRS and FC in 30 participants in each drug condition.

#### Statistical analysis

Statistical analysis of interrater reliability, outcome variables of the creativity tasks, subjective effects, and relative glutamate concentrations were conducted in IBM SPSS Statistics 24. To assess the interrater reliability, intra-class correlation coefficients (ICC) and their 95% confidence intervals were calculated based on a mean-rating (*k* = 2), consistency, two-way random-effects model for relevant PCT- and AUT-dependent variables. Reliability derived from ICC values can range from poor (<0.5), to moderate (0.5–0.75), good (0.75–0.9), and excellent (>0.90)^66^.

Baseline-change scores of the AUT and PCT outcome measures followed normal distributions, and the variances between groups were assumed to be similar. Outcome measures entered separate mixed model ANOVAs consisting of the between-subject factor treatment (psilocybin or placebo) and the within-subject factors time (acute and follow-up), and construct (the dependent outcome variables of the PCT and AUT). In case of a treatment × time × construct interaction, independent samples *t* tests were conducted in order to assess differences between groups. Nonparametric Mann–Whitney *U* tests were used to assess treatment group differences in ratings of the 5D-ASC subscale “insightfulness”, and differences in relative glutamate concentrations, as these data points did not meet the assumption of normality. The alpha criterion level of significance of all aforementioned tests was set at *p* = 0.05 (two-tailed).

For the assessment of within-network FC, the unthresholded ICA component images were compared between placebo and drug conditions (two sample *t* test). Parametric statistics were used (voxel threshold *p* < 0.001 uncorrected, cluster threshold *p* < 0.05 cluster size, false discovery rate (FDR) corrected, two-sided).

For the assessment of between-network FC, ICA components were thresholded at *z* > 2 and binarized. The mean of the time series was extracted for every resulting ROI. Time courses between all RSNs were then compared for both conditions using bivariate correlations. The resulting correlation coefficients were compared between placebo and drug conditions (two sample *t* test). Results were corrected for multiple comparisons using FDR.

Canonical correlations^67^ were conducted to evaluate the association between changes in variables that showed a significant treatment effect, including (i) outcome measures of the PCT and AUT, (ii) ratings of the 5D-ASC dimension, insightfulness, (iii) within- and between-network resting-state FC, using extracted connectivity strength (beta) values, and (iv) relative glutamate concentration levels in the mPFC and hippocampus. Variables were separated into two sets; set 1 included the cognitive and subjective measures of creativity [(i) and (ii)] and set 2 included biological variables as predictors [(iii) and (iv)]. Canonical correlations were chosen as this approach assesses the relationship between two multivariate data sets, allowing investigation of variables that may have multiple causes and effects, while also reducing the potential of type 1 error^67^. An iterative imputation approach was performed to fill in missing data points, when applicable.

## Results

### Demographic variables, psilocin blood serum concentrations, subjective state, and relative glutamate concentrations

Demographic information, mean (SE) psilocin blood serum concentrations, ratings on the 5D-ASC, and relative glutamate concentrations are all previously published elsewhere^51^ and briefly summarized here.

The psilocybin group (*n* = 30) and the placebo group (*n* = 30) did not differ in respect to demographic variables, such as gender, age, and drug use history. Psilocin blood serum concentrations reached a peak at 80 min post administration (15.61 ± 1.66 ng/mL), while participants were in the MRI scanner, and were 12.86 ± 1.13 ng/mL during creativity task completion (150 min post). Administration of psilocybin was also associated with increased ratings on all (sub)dimensions of the 5D-ASC, including the dimension of interest for this study, “insightfulness” (*n* = 60, *U* = 44.5; *p* ≤ 0.001, *d* = 1.78). Finally, compared to placebo, relative glutamate concentrations were significantly higher in the mPFC (mean ± SE; psilocybin: 1.23 ± 0.02, *n* = 24; placebo: 1.14 ± 0.02, *n* = 28; *U* = 200.50, *p* = 0.01, *d* = 0.80) and significantly lower in the hippocampus (psilocybin: 0.77 ± 0.03, *n* = 21; placebo: 0.88 ± 0.03, *n* = 25; *U* = 163.50, *p* = 0.03, *d* = 0.69).

### Creativity tasks

The ICC for originality was 0.95 on the PCT and 0.91 on the AUT, indicating excellent reliability. The ICC for all outcome variables, including those not within the scope of this study can be found in Table S1.

#### Picture concept test

**Fig. 1:**
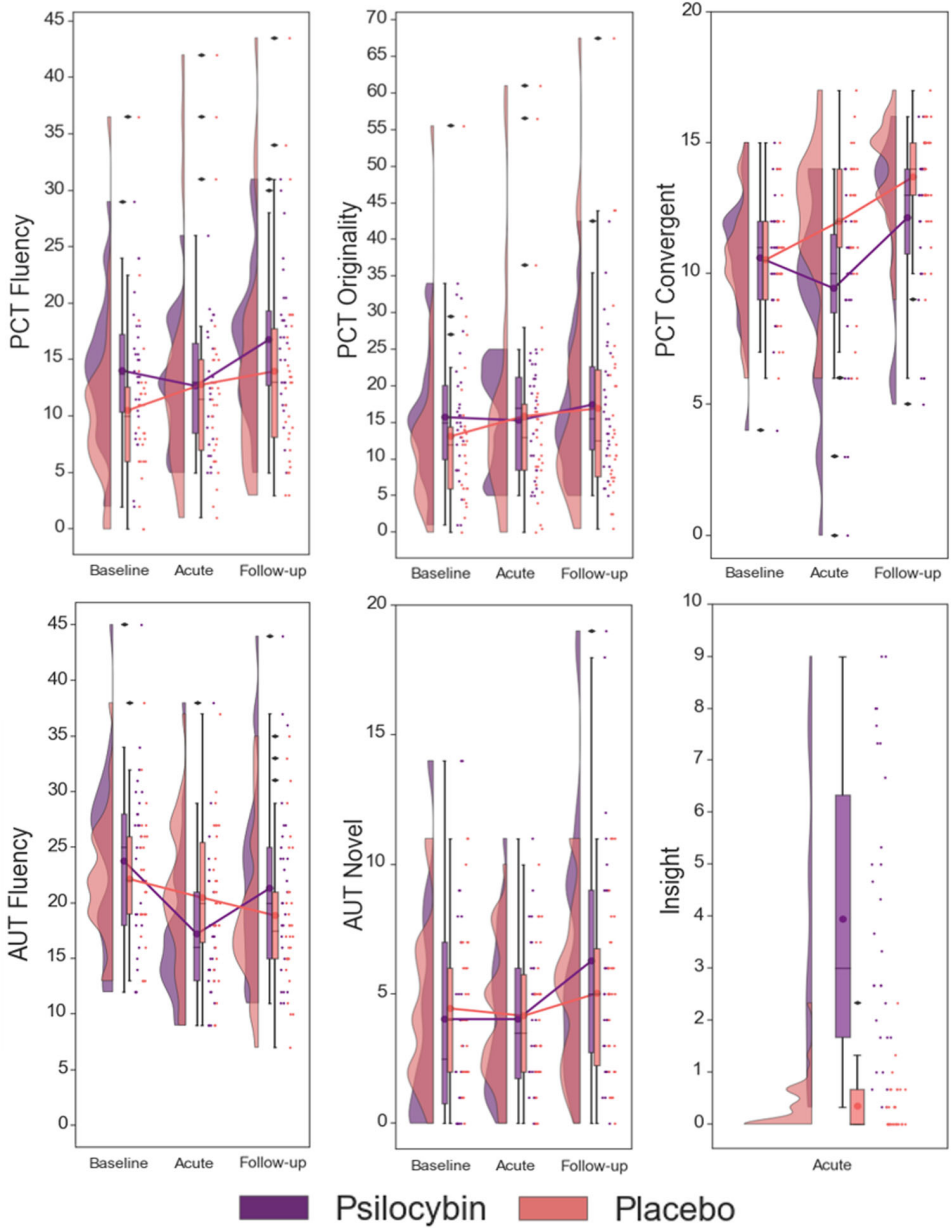
Raincloud plots displaying scores on measures of creativity, which demonstrated significant differences between treatment groups. The plot consists of a probability density plot, a boxplot, and raw data points. In the boxplot, the line dividing the box represents the median of the data, the ends represent the upper/lower quartiles, and the extreme lines represent the highest and lowest values excluding outliers. The code for raincloud plot visualization has been adapted from Allen et al.^108^.

Psilocybin influenced performance on the PCT in a time- and construct-dependent manner, as evidenced by a significant treatment × time × construct interaction (*F*_3,47_ = 4.53, *p* = 0.007; psilocybin *n* = 25, placebo *n* = 26). Compared to placebo, psilocybin acutely decreased CT (*d* = 0.85), and measures of DT including fluency (*d* = 0.84), and originality (*d* = 0.65). At the 7-day follow-up, CT was still significantly decreased when comparing psilocybin to placebo (*d* = 0.60; Table 1 and Fig. 1).

**Table 1:** Mean (SE) and independent *t* test results (psilocybin vs placebo) of all dependent outcome variables on the AUT and PCT.

Variable	Acute					Long term				
	Psilocybin	Placebo	*T* value	*P* value	Cohen’s *d*	Psilocybin	Placebo	*T* value	*P* value	Cohen’s *d*
AUT										
Fluency	−6.55 (1.13)	−1.89 (1.02)	−3.06	<0.01*	0.80	−2.44 (0.89)	−3.62 (0.96)	0.89	0.37	0.23
Originality	−1.62 (0.97)	−0.48 (1.22)	−0.73	0.47	0.19	−0.14 (0.83)	−0.72 (0.77)	0.51	0.61	0.13
Ratio	0.07 (0.03)	0.02 (0.05)	0.81	0.42	0.21	0.04 (0.03)	0.05 (0.04)	−0.34	0.73	0.10
Novel	−0.31 (0.76)	−0.21 (0.49)	−0.11	0.91	0.03	2.25 (0.76)	0.45 (0.53)	1.96	0.05*	0.52
PCT										
Fluency	−2.73 (1.81)	4.19 (1.40)	−3.02	<0.01*	0.84	4.07 (1.24)	5.25 (1.39)	−0.63	0.53	0.17
Originality	−0.711 (1.65)	4.54 (1.49)	−2.36	0.02*	0.65	1.59 (1.32)	5.31 (1.52)	−1.84	0.07	0.50
Ratio	0.10 (0.035)	0.09 (0.063)	0.04	0.96	0.01	−0.04 (0.03)	0.06 (0.06)	−1.53	0.13	0.42
Convergent	−1.00 (0.76)	1.61 (0.37)	−3.15	<0.01*	0.85	1.67 (0.57)	3.36 (0.49)	−2.24	0.03*	0.60

*Statistical significance at the *p* = 0.05 level.

#### Alternative uses test

Psilocybin influenced performance on the AUT in a time- and construct-dependent manner, as evidenced by a significant treatment × time × construct interaction (*F*_3,53_ = 4.50, *p* = 0.007; psilocybin *n* = 28, placebo *n* = 29). Compared to placebo, psilocybin acutely decreased fluency (*d* = 0.80). Conversely, compared to placebo psilocybin significantly increased scores of novelty (*d* = 0.52) at the follow-up (Table 1 and Fig. 1).

### Resting-state networks

After quality control of the imaging data, the final sample consisted of 22 participants in the psilocybin group and 26 in the placebo group. There were no significant differences between groups in regards to head motion parameters. See Supplementary Material for exclusion criteria and assessed differences between groups.

#### Independent component analysis

There was a good agreement between the components identified in our analysis and the templates provided by Smith, et al.^62^. The results of this full analysis are published elsewhere^51^. For the purpose of this study, we focused on the frontoparietal network 1 (*r* = 0.50) and 2 (*r* = 0.47) and the DMN, which was split into subcomponents (anterior: *r* = 0.34 and posterior: *r* = 0.52), as has already been observed in multiple studies^68,69^. The anterior SN (*r* = 0.39) was identified via the component of Shirer et al.^70^ as an SN template is not identified in ref. ^62^.

#### Within-network connectivity

**Fig. 2:**
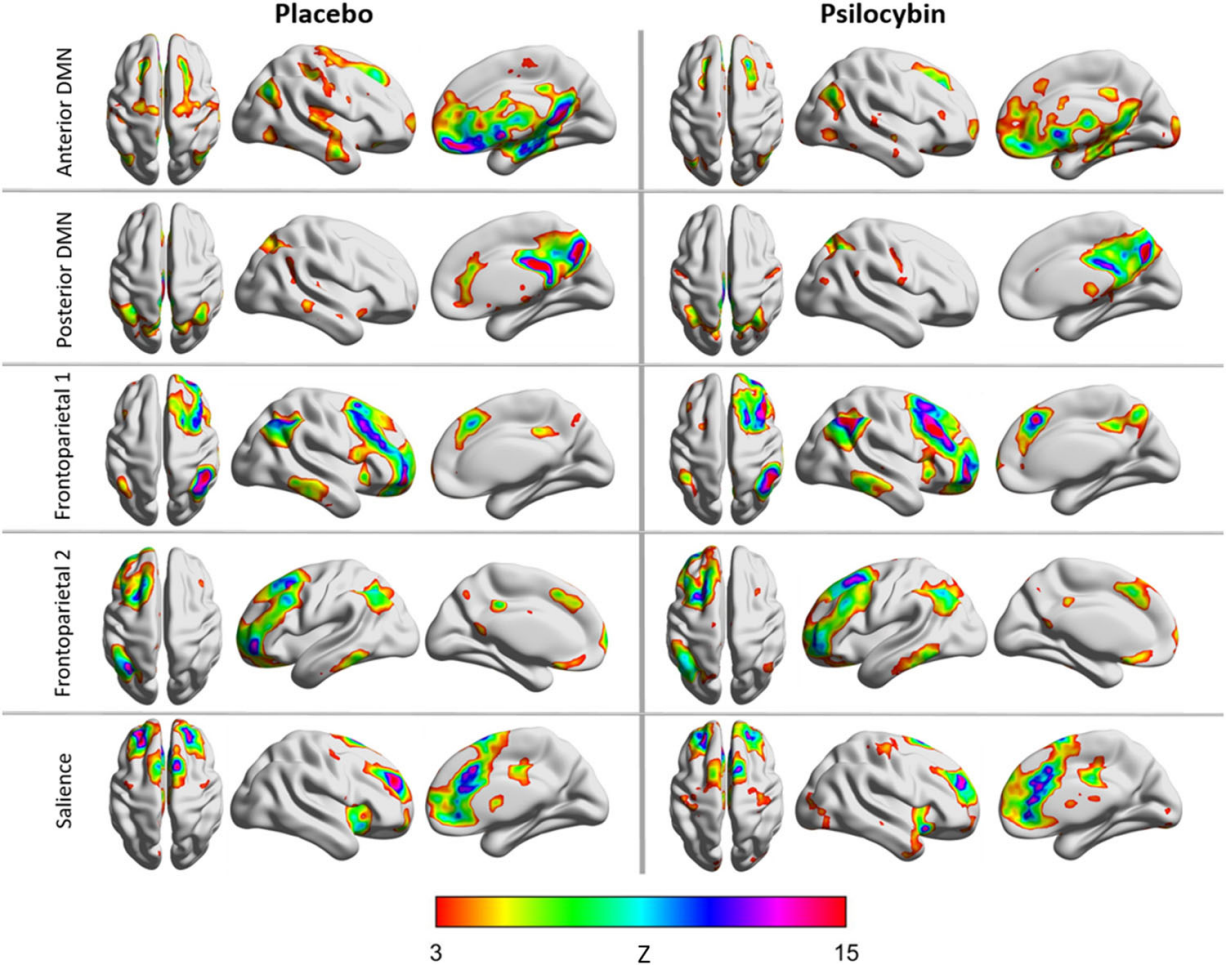
Resting-state networks implicated in creative cognition, for each group (placebo and psilocybin). The anterior and posterior DMN demonstrated significant differences in within-network functional connectivity between groups.

Within the respective network, significantly less coactivation under the drug condition relative to placebo was found in both subcomponents of the DMN (anterior and posterior). FC within the FPN and SN did not differ between psilocybin and placebo (Fig. 2).

#### Between-network connectivity

**Fig. 3:**
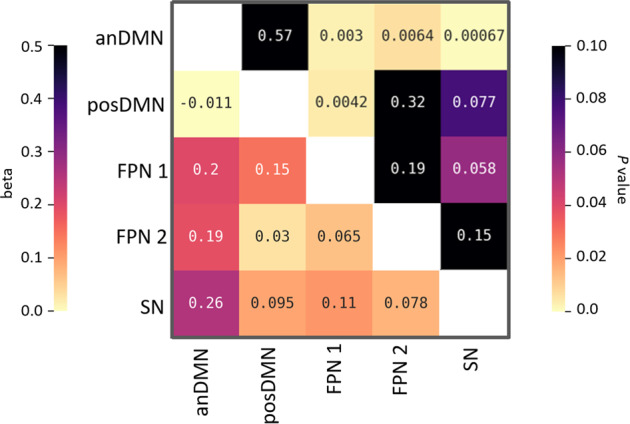
Correlation matrix illustrating increased functional connectivity between resting-state networks under psilocybin compared to placebo (psilocybin > placebo). The left half of the matrix refers to the beta values, and the right half of the matrix refers to the *p* value.

Widespread increases in between-network FC were observed under psilocybin compared to placebo, with significant increases between the anterior and posterior DMN and the FPN 1, the anterior DMN and the FPN 2, and between the anterior DMN and the SN (Fig. 3).

### Assessing biological predictors of creativity

A canonical correlation analysis was conducted using the eight biological variables as predictors of the eight creativity outcome measures to evaluate the multivariate shared relationship between the two variable sets. The analysis yielded seven functions with squared canonical correlations (*R*_c_^2^) of 0.553, 0.483, 0.165, 0.135, 0.075. 042, and 0.001 for each successive function. The full model across all functions was statistically significant *F*(56,193.79) = 1.48, *p* = 0.027), explaining 85.3% of the variance. From this model, the first three functions were considered noteworthy in the context of this study, explaining 55.3%, 48.3%, and 16.5% of the variance, respectively.

Table 2 presents the standardized canonical function coefficients, the structure coefficients (*r*_s_), and the squared structure coefficients (*r*_s_^2^*)* for each function, as well as the communalities (*h*^2^) across the functions for each variable (for an in-depth explanation and interpretation of canonical correlations and associated terminology, the reader is referred to Sherry and Henson^67^). Function 1 indicated that the dominant contributor was ratings of insight and the secondary contributor was long-term changes in novel responses, with posterior DMN and anterior DMN FC being the dominant predictors, and secondary predictors being hippocampal glutamate levels. Overall, this function suggests that the strongest predictor of increasingly higher feelings of insight and (positive) changes in long-term novelty, were lower levels of within-network DMN FC acutely.

**Table 2:** Canonical solution for biological variables predicting creativity variables for all functions.

	Function 1			Function 2			Function 3			
Variable	Coef	*r*_s_	*r*_s_^2^ (%)	Coef	*r*_s_	*r*_s_^2^ (%)	Coef	*r*_s_	*r*_s_^2^ (%)	*h*^2^(%)
AUT fluency	0.10	−0.40	15.68	−0.14	−0.04	0.12	0.08	−0.21	4.54	20.34
PCT fluency	−0.02	−0.41	16.73	1.45	−0.02	0.06	0.44	0.32	10.24	27.03
PCT originality	−0.17	−0.45	20.70	−1.66	−0.49	23.72	−0.33	0.22	4.80	49.22
PCT convergent	0.11	−0.38	14.67	0.11	0.08	0.61	0.75	0.78	61.00	76.27
Insight	0.83	0.89	79.21	−0.39	−0.21	4.37	0.62	0.29	8.12	91.70
PCT convergent long term	−0.32	−0.44	19.71	−0.02	0.03	0.10	0.22	0.58	33.52	53.33
AUT novelty long term	0.24	0.47	22.37	0.33	0.38	14.75	−0.21	−0.21	4.33	41.44
*R*_c_^2^			55.3			48.3			16.5	
AnDMN	−0.67	−0.80	63.36	−0.94	−0.17	3.03	−0.18	0.04	0.17	66.56
PosDMN	−0.30	−0.72	52.13	0.92	0.56	30.80	−0.38	−0.15	2.22	85.15
anDMN–SN	0.10	0.34	11.70	0.13	−0.20	3.88	−0.38	−0.53	28.20	43.77
anDMN–FPN 1	0.25	0.28	7.90	−0.39	−0.36	12.74	−0.40	−0.67	44.76	65.40
posDMN–FPN 1	0.07	0.24	5.86	0.12	0.15	2.16	−0.42	−0.70	49.14	57.16
anDMN–FPN 2	−0.53	0.11	1.10	−0.42	−0.35	12.53	−0.14	−0.46	21.44	35.07
Hippocampal glutamate	−0.22	−0.49	24.30	0.18	0.21	4.28	−0.10	−0.30	8.94	37.53
mPFC glutamate	0.21	0.37	13.62	0.24	0.03	0.07	−0.28	−0.30	9.18	22.87

*r*_s_ > |0.45| and *h*^2^ > 45% are underlined and deemed valuable contributors.

Function 2 indicated that the dominant contributors were the acute changes in originality, with the dominant predictor being posterior DMN FC, suggesting that the strongest predictor of larger (negative) changes in acute originality were lower levels of within-network posterior DMN FC.

Function 3 indicated that the dominant contributors were the acute and long-term changes in CT, whereas the dominant predictors were FC between both components of the DMN and the FPN. This function suggests that the strongest predictor of larger (negative) changes in acute and long-term CT were higher levels of between-network FC between the DMN and the FPN.

## Discussion

The present study demonstrates the first modern-day attempt to experimentally assess the (sub)acute effects of a psychedelic on divergent and CT, the two constructs of creative thinking. Findings demonstrate that psilocybin induces a time- and construct-related differentiation of effects on creative thinking; whereas CT and aspects of DT were shown to decrease during the psychedelic state, an aspect of DT was shown to increase 7 days later compared to placebo. Acutely, individuals under psilocybin also reported higher subjective ratings on a measure akin to spontaneous creative thinking. Analyses indicated that alterations in creative thinking were further predicted by acute alterations in RSFC. Whereas disintegration of the DMN predicted both higher scores in spontaneous creative thinking and long-term increases in novelty of generated ideas, increased connectivity between the DMN and the FPN predicted decreases in acute and long-term CT.

### Acute and persisting effects of psilocybin on divergent thinking

Contrary to our hypothesis, results indicate that aspects of DT, or the idea generation phase of the creative process, are impaired during the acute psychedelic state. Namely, participants generated less ideas and associations when under psilocybin compared to placebo, as indicated by significantly lower fluency scores on the AUT and the PCT, respectively. Originality scores were also lower after psilocybin on the PCT; however, when controlling quality for quantity (how many responses were given; ratio), there were no significant differences. Thus, when assessing how unique generated responses were, there was no significant difference between treatments. That said, individuals under the influence of psilocybin reported significantly higher ratings of “insightfulness”, a construct conceptually related to spontaneous creative thinking (e.g., “I had insights into connections that had previously puzzled me” and “I had very original thoughts”). Thus, overall, results suggest psilocybin acutely reduces an individual’s volume of new ideas and associations, without modulating quality, in response to goal-directed creativity tasks, while increasing feelings of spontaneous creative insights.

In line with our hypothesis, results indicate an increase in aspects of DT after the acute drug effects have worn off. Namely, 7 days after psilocybin administration, participants generated a higher quantity of novel ideas for uses of an everyday object on the AUT compared to placebo. That said, there were no differences in amount of generated ideas or associations in total, or how original responses were. It should also be noted that the effect size on number of novel ideas was moderate (*d* = 0.52), however, is in accordance with other studies that have found persisting changes in aspects related to DT at various time points relative to consumption of a range of psychedelic drugs (see Aday et al.^71^ for a review).

### Acute and persisting effects of psilocybin on convergent thinking

CT can be seen as the second phase in the creative process, focused on evaluating possibilities generated during DT, and narrowing them down to a logical solution^4^. In line with previous studies suggesting that psychedelics decrease conventional, logical thinking^16,17,20^, we found an acute decrement in CT when comparing psilocybin to placebo. When comparing the two treatment groups, we also found that the psilocybin group performed significantly worse compared to placebo at follow-up. However, it should be noted that although there is a significant difference when looking between groups, when looking within groups, there is an increase in performance on the CT in the placebo group when comparing baseline to acute and follow-up, and an increase in performance in the psilocybin group when comparing baseline to the follow-up. Thus, although measures were taken to reduce potential learning effects on the PCT, including administering three different versions of the task, and withholding feedback on performance, our data indicate that this could be taking place. The finding that psilocybin acutely blocked this learning effect adds to the literature that psychedelics disrupt associative learning^72^ and impair executive functioning^73^; effects that have been proposed to be mediated via agonistic activation at the 5-HT_2A_ receptor^73^, and potentially due to impaired attentional performance resulting from an inability to ignore task-irrelevant stimuli^74,75^. This suggestion of learning effects also brings into question findings from recent naturalistic studies that found persisting enhancements in CT on the PCT after intake of a psychedelic^21–23,76^, and emphasizes the importance of a placebo control.

### Do psychedelics enhance creative thinking?

Results of our study suggests that psychedelics do not enhance creative thinking per se, but rather mediate changes in particular constructs of creative thinking, in a time-dependent manner. Interestingly, these constructs could be interpreted in two different ways, either emphasizing a discrepancy between objective vs subjective creative performance, or highlighting the difference between spontaneous vs deliberate creative cognition.

In regards to the former, our data could suggest that psilocybin acutely impairs the idea generation and evaluation phase of creative thinking, while enhancing the feelings of quality of generated ideas (measured via the subjective questionnaire of insightfulness), despite lack of objective evidence. This discrepancy between a subjective sense of enhanced creative thinking, and objective assessments of creativity is a mismatch that is also found in the early psychedelic creativity literature^10^. Increased feelings of insight, changes in affect, and attribution of meaning to previously neutral stimuli are recognized as common acute effects of psychedelic drugs^77–82^, and thus it is plausible that reports of increased creativity during the acute psychedelic state are due to a subjective sense of creativity enhancement that does not match the objective quality^1^. However, 1 week later this discrepancy appeared to dissipate, in that it was objectively found that the idea generation phase is enhanced, with a higher number of novel ideas reported after psilocybin.

An alternative interpretation of our data could arise from the difference between two processing modes of creative cognition, deliberate vs spontaneous^48^. The deliberate processing mode is initiated when one is focused on meeting explicit task demands, such as in a guided, timed situation (as is the case with our tasks), and is characterized as being more attention-demanding, searching along structured and rational belief systems in order to reach a goal^48^. In contrast, spontaneous insights tend to occur in a mental state characterized by unrestrained cognition and defocused attention, and tend to be more random, unfiltered, and bizarre^48^; a mental state almost synonymous with the characterization of the psychedelic state^83^. Thus, this discrepancy between acute increases in feelings of insight, but decreased number of ideas, could suggest that psychedelics acutely increase the potential for spontaneous creative thought, while decreasing the potential for deliberate creative cognition. As it has been suggested that productive creative cognition may likely be facilitated by a balance between spontaneous insights and controlled deliberate processing^47^, our data suggest that psychedelics disrupt this balance acutely, whereas subacutely this balance is restored, with deliberate creative cognition potentially even enhanced, as evidenced by an increased number of novel ideas one week later. A post hoc correlation analysis also indicates that there may be a relationship between acute increases in spontaneous creativity, and long-term increases in idea generation, as a significant positive correlation was found between the two (see Supplemenatary Results).

### Biological predictors of creative thinking

As we found that psychedelics can mediate changes in particular constructs of creative thinking, it suggests that they could be a novel tool to investigate underlying neural mechanisms of the creative process. Thus, canonical correlations were conducted to predict performance on DT and CT from acute biological parameters hypothesized to underlie creative cognition, including within- and between-network FC of the DMN, FPN, and SN, and glutamate concentrations in the mPFC and the hippocampus.

Decreased integrity of the DMN was found to be the strongest predictor in regards to increased feelings of acute subjective spontaneous creative thinking (insightfulness). In general, this is in line with previous work that has implicated the DMN in subjective effects representing unconstrained cognition during the psychedelic state^1,43,81,84^, as well as the spontaneous processing mode in creative cognition^47^. Changes in DMN activity and integrity have been repeatedly found during the acute psychedelic state^33,34,36,81,85^, and a disruption of coupling between the medial temporal lobes (MTLs) and the DMN^29^, has been particularly implicated in explaining some of the key subjective effects of the drugs, including feelings of insightfulness^81^, and an altered sense of self and reality^84,86^. In addition, DMN and MTL activity have both been implicated in dreaming^87^, a state neurophenomenologically similar to the psychedelic state^1,83,88,89^ in that both are characterized by experience of bizarre cognitive phenomena, illogical transitions between thoughts, and vivid sensorimotor imagery^90^. Interestingly, dreaming has been suggested to be the most extreme form of the spontaneous processing mode in creative cognition^48^. Thus taken together, our data suggest that the psychedelic state may increase the potential for spontaneous creative thought, and does so via disruption of DMN integrity. In addition, we found that decreased glutamate in the hippocampus was a secondary predictor in increased ratings of insight. As it has been suggested that MTL activity is a crucial driver of the DMN^29^, and the relationship between the two has been implicated in the psychedelic and related states, it could be suggested that glutamatergic activity of hippocampal regions could underlie this desynchronization. The involvement of the hippocampus in creative cognition is also interesting to speculate on, as it has been found that past knowledge about a problem can actually be detrimental to solving it, constraining potential ideas to what is already known, and thus is suggested to be a disadvantage of the deliberate mode of creative cognition, and an advantage of the spontaneous mode(see Dietrich^48^ for a review).

In regards to deliberate, task-based creative thinking, the finding that decreased within-network DMN FC predicted both an acute decrease in scores of originality, and a long-term increase in generation of novel ideas may seem counterintuitive, however, actually is in line with previous work. Specifically, given the suggested role of the DMN in underlying the idea generation process of DT, an acute decrement in DMN FC resulting in an acute decrement in DT performance on a creativity task would be expected. In addition, previous work has found that while psychedelics decrease within-network DMN FC acutely, they increase DMN integrity subacutely^41–44^, potentially via a neuroplastic effect on brain network function^44,91,92^. Thus, it could be suggested that the latter prediction seen here is reflecting the subsequent psilocybin-induced increase in DMN FC that could facilitate an increase in novel ideas. Such findings add support to the suggestion that novel idea generation is mediated via DMN network FC^1,25–27^, and also suggest that current frameworks conceptualizing the impact of the psychedelic state on creativity could be adapted to include both acute and persisting effects of the drug^1^.

Finally, levels of acute connectivity between both subcomponents of the DMN and the FPN were found to inversely predict acute and long-term changes in CT performance. Namely, increasingly positive DMN–FPN connectivity predicted increasingly worse CT performance relative to baseline. Although usually anticorrelated^93,94^, previous studies have found that, during a task, higher dynamic DMN–FPN connectivity predicted better performance in areas, such as creative cognition, cognitive flexibility, attention, and learning^25,26,95–98^. In regards to creative cognition in particular, it has been suggested that during the creative process, the degree of FPN involvement increases as the need for more deliberate constraints on thought content (in order to reach, e.g., a goal) increases^1^. Further, it has been suggested that productive creative cognition is facilitated by a balance between spontaneous and controlled processing^48^, both of which may be experienced concurrently via coupling between the DMN and task positive networks like the FPN^47^. Thus, the association between DMN–FPN connectivity and CT is in line with previous work implicating DMN–FPN dynamics in the idea evaluation phase of the creative process. That said, it may then be hypothesized that increased DMN–FPN coupling would result in better performance on a task of CT. However, the aforementioned evidence stems from measurements taken while individuals were performing a task. Instead, it has been found that higher resting-state FPN–DMN dynamics relates to poorer performance on tasks of cognitive flexibility, and has also been found in states characterized by poorer attention, including during the psychedelic state^38^, and in patients with ADHD^99^. Thus increased DMN–FPN between-network connectivity at rest could be reflecting the potential of either intrusion of, or insufficient suppression of, the DMN during a task. As increased resting-state DMN–FPN connectivity has also been found in patients characterized by poorer attentional abilities, and psychedelics have been found to disrupt executive functions potentially via impairment of attention^74,75^, this could provide a biological basis for how psychedelics impair executive functioning, and could explain how psilocybin blocked the suggested CT learning effect seen during our study.

Although the role of the mPFC has been strongly highlighted in creative cognition^48^, glutamate concentrations in this area did not play a significant role in the canonical model. This could suggest that the involvement of the mPFC is due to its role in integration and interaction of distributed brain areas operating in large-scale networks^100^, rather than underlying neural activity in the specific brain area. In addition, although the connectivity between the DMN and the SN has been implicated in the creative process, in that the SN has been suggested to facilitate the shift of ideas from the DMN to the FPN^25–27^, we did not find that this strongly predicted any outcome measures of creative thinking. The latter may be due to the fact that we are correlating network activity at rest, with behavior measures taken outside of the scanner, emphasizing a limitation of this study. Thus, although consistent with the previously discussed literature, it is important to note that discrepancies have been found between state and trait network connectivity and creative performance^101,102^. In light of this, results should be taken as preliminary, and future studies assessing the neurobiological effects of psychedelics on creativity should employ creativity tasks during scanning.

## Conclusion

In conclusion, this study found that psilocybin induces a time- and construct-related differentiation of effects on creative thinking, suggesting that psychedelics could be a novel tool to investigate underlying neural mechanisms of the creative process^1,24^. In addition, these findings add some support to the historical claims that psychedelics can influence aspects of the creative process, reducing conventional, logical thinking, and giving rise to novel thoughts, but emphasizes the distinction between spontaneous and deliberate creative cognition, as well as acute and persisting effects of the drug.

These distinctions are of particular importance, as psychedelics are currently being investigated to treat a number of mental health disorders^103^, characterized by rigid, inflexible thought patterns^29,104,105^. Thus, it could be suggested that the ability of psilocybin to acutely decrease CT and increase spontaneous DT, while subacutely enhancing more goal-directed DT, could aid in the therapeutic process by opening up a window of opportunity where therapeutic interventions could prove more effective. Namely, while under the influence of a psychedelic, rigid thought content (CT) could be decreased, while unguided, spontaneous thoughts may give rise to new insights and perspectives of previous events and current problems^19,106,107^. Subacutely, patients may then be able to integrate these insights with a therapist, and come up with new, more effective strategies that facilitate adaptive interpretation and coping abilities^8^. Nevertheless, future studies should be employed in clinical populations in order to assess this proposal, as well as the underlying neurobiological mechanisms.

## Supplementary information

Supplemental information
