# Supplementary material for: Spontaneous and deliberate creative cognition during and after psilocybin exposure
Source: Transl Psychiatry. 2021 Apr 8;11:209. doi: 10.1038/s41398-021-01335-5 (PMC8032715; doi:10.1038/s41398-021-01335-5)
Supplement: Supplementary file 1 — Supplemental information [file 41398_2021_1335_MOESM1_ESM.docx]

**Supplementary Information for**

spontaneous and deliberate creative cognition during and after psilocybin exposure

***Corresponding author**

Email: natasha.mason@maastrichtuniversity.nl

**Manuscript details**

Word count text: 2760

Display Items (Figures and Tables): 1

| Table S1. ICC interrater reliability for all dependent outcome variables on the creativity task, which were scored by 2 independent raters | | | | | | | |
| --- | --- | --- | --- | --- | --- | --- | --- |
|  | Variable | ICC | 95% CI | F | Df1 | Df2 | p |
| AUT | Originality | .909 | .867-.941 | 10.964 | 57 | 285 | .000 |
|  | Remote | .884 | .830-.925 | 8.607 | 57 | 285 | .000 |
|  | Clever | .879 | .824-.922 | 8.292 | 57 | 285 | .000 |
|  | Creative | .901 | .855-.936 | 10.060 | 57 | 285 | .000 |
| PCT | Originality | .947 | .922-.966 | 18.921 | 52 | 260 | .000 |

**Methods**

**Participants.** Participants were recruited through advertisements around Maastricht University and internet forums in the Netherlands. Inclusion criteria were: age, 18-40 years; previous experience with a psychedelic drug, but not within the past 3 months; normal weight, body mass index between 18 and 28 kg/m2; free from psychotropic medication; good physical health, including absence of major medical, endocrine, and neurological conditions; and written informed consent. Exclusion criteria were: history of drug abuse or addiction; pregnancy or lactation; health issues including hypertension (diastolic >90 and systolic >140), cardiac dysfunction, and liver dysfunction; current or history of psychiatric disorders; previous experience of serious side effects to psychedelics; and MRI contraindications. Before inclusion, subjects answered medical questionnaires about their health and drug use, and were screened and examined by a study physician, who checked for general health, conducted a resting ECG, and took blood and urine samples in which hematology, clinical chemistry, urine, and virology analyses were conducted. Flow diagram of participant recruitment and analysis can be found in Figure S1. Participant demographic data can be found in Table S1 of the previous publication[^1^](#_ENREF_1).

Psilocybin (powder) was obtained from GH Pharm GmbH, Frankfurt, Germany. A permit for obtaining, storing, and administering psilocybin was obtained from the Dutch Drug Enforcement Administration. Participants were financially compensated for their participation in the study.

**Randomization and blinding.** An experimenter who was not responsible for treatment randomization or preparation, recruited all participants. A separate experimenter, who did not come in direct contact with the subjects, Allocated treatment in a completely random order. This latter experimenter was also responsible for preparing the treatment (psilocybin or placebo), and giving the treatment to the data collector in a closed cup. The closed cup ensured that neither the data collector or the participant would be unblinded as to what treatment the participant was receiving. Only following completion of all data collection, was the study fully unblinded.

**Procedure.** Participants were familiarized with the test day procedures on a separate training day prior to the treatment conditions, where they also completed the baseline creativity tests. Participants were instructed to refrain from drug use, including psychedelic drugs (≥ 3 months), MDMA/ecstasy (≥ 14 days), alcohol (≥24 hours), and all other drugs of abuse (≥7 days) prior to their first testing day, and to remain sober until completion of the follow-up testing day (7 days later). Additionally, participants were asked to refrain from caffeine and nicotine use the day of the test day.

On arrival of a test day, absence of drug and alcohol use was assessed via a urine drug screen and a breath alcohol screen. An additional pregnancy test was given if participants were female. If all tests were found to be negative, participants were allowed to proceed, and a venal catheter was placed, in order to take blood samples throughout the testing day. Before administration of treatment, a baseline blood sample was taken and baseline vital signs (blood pressure and heart rate) were measured. After measurements, the treatment was administered orally, in a closed cup containing bitter lemon (placebo) or bitter lemon and psilocybin (powder; 0.17 mg/kg psilocybin). Bitter lemon was used in order to conceal any potential taste of psilocybin. After 40 minutes, participants were placed in the MRI scanner, where resting state scans and magnetic resonance spectroscopy were performed throughout a 1 hour time window. When they were taken out of the MRI scanner, they returned to the laboratory room where they completed the creativity tests. At the end of the test day (approximately 6 hours after treatment administration), participants were asked to complete measures of retrospective subjective high. Participants stayed under supervision until the testing day was complete, and the researcher deemed they were fit to go home.

Participants then returned 7 days later, and were screened again for drug and alcohol use, and if applicable pregnancy, via the aforementioned methods. If the tests were found to be negative, they were allowed to proceed and complete the creativity tests. If they were positive, participants were sent home and excluded from the study (n=0).

**Picture Concept Task.** The PCT consists of 17 stimuli, each containing between 4 and 12 color pictures shown in a matrix of 2 × 2, 2 × 3, 3 × 3, or 3 × 4. Participants were given 30 seconds per stimulus to find an association between one of the pictures in each row. Specifically, they were asked first to provide the correct solution, as there is only one correct answer. The number of correct answers served as the dependent measure of convergent thinking. In order to assess divergent thinking, participants were asked to provide as many alternative answers as possible. This is the regular instruction included in measures of divergent thinking, and it is used to calculate several parameters—i.e., fluency, originality, and the ratio of both—which reflect quantity and quality of divergent thinking. Fluency is defined as the number of alternative associations. The second parameter (i.e., originality) is calculated by evaluating the originality of the alternative association relative to those provided by all other participants in a session. Alternative answers that were uniquely reported by a single participant received an originality score of 2, answers that were shared with a single participant were valued as 1, and answers that were shared by three or more participants were rated zero. Originality was the sum of the originality points. In addition, the ratio of originality and fluency was calculated, to correct originality for the number of responses that were generated. When a participant would give two responses of ‘medium’ originality, which is worth one point, and another participant would give only one highly original response, which is worth two points, Originality would be awarded with two in total for both. Even though the scores are the same, the quality is not. Ratio reflects this difference in quality with the first participant in the example having Ratio ‘1’ and the second participant Ratio ‘2’.

**AUT.** Three different versions of the AUT were also given to participants. On version A, participants were asked to come up with as many alternative uses as possible for “pen” and “newspaper”, version B included the two words “towel” and “bottle”, and version C included “brick” and “shoe”. Here originality scores were calculated as 2 points if the answer was given by less than 1% of the participants, and 1 point if the answer was given by less than 5% of the participants. The AUT can also be scored in regards to additional outcome measures[^2^](#_ENREF_2), which we decided not to include in this study in order to be as succinct as possible between creativity tasks. These outcome variables include remoteness of association (remote), cleverness of the response (clever), and perceived creativity of the response (creative), flexibility, and elaboration. The ability to generate a diversity of responses ‘Flexibility’ was assessed by clustering the responses into categories; the sum of categories was the dependent variable. The amount of detail in the responses, ‘Elaboration’ was scored by summating the number of details.

**Questionnaires**

**5-Dimensional Altered States of Consciousness Rating Scale.** The 5D-ASC is a 94-item self-report scale that assesses the participants’ alterations from normal waking consciousness[^3^](#_ENREF_3). The participant is asked to make a vertical mark on the 10-cm line below each statement to rate to what extent the statements applied to their experience in retrospect from “No, not more than usually” to “Yes, more than usually.” The 5D-ASC contains the 5 key dimensions, including anxious ego dissolution, visual restructuralization, auditory alterations, reduction of vigilance, and oceanic boundlessness; which can be broken down into 11 subscales consisting of experience of unity, spiritual experience, blissful state, insightfulness, disembodiment, impaired control and cognition, anxiety, complex imagery, elementary imagery, audio-visual synesthesia, and changed meaning of percepts

**Blood**

All samples were centrifuged and serum was frozen at -20°C, and kept in the dark until analysis. Analysis of psilocin in serum was performed according to [Martin, et al. ^4^](#_ENREF_4). Serum (200 µl) was extracted with 1 ml of ethyl acetate after addition of phosphate buffer pH 9, 20 ng psilocine-d_10_ and 10 µl of 0.1 M ascorbic acid for stabilization. The organic phase was evaporated and reconstituted with 100 µl of 0.1 % formic acid/acetonitrile (80:20, v/v). The analysis of 2 µl was performed on an Agilent (Waldbronn, Germany) LC-MS/MS system consisting of a 1290 Infinity Liquid Chromatograph coupled via JetStream Electrospray Interface (ESI) to a G6460A Triple Quadrupole Mass Spectrometer. Analytes were separated on a Kinetex® 2.6 µm XB-C18 100 Å LC column (100 x 2.1 mm) plus corresponding guard column from Phenomenex (Aschaffenburg, Germany) at 30 °C. Gradient elution at a flow rate of 0.5 ml/min using 0.01% formic acid containing 5 mM ammonium formate (A) and acetonitrile containing 0.1 % formic acid (B) started with 5 % B, increased to 95 % B during 4 min and was held for 2 min. Source parameters were: gas temperature 300 °C, gas flow 11 l/min, nebulizer 45 psi, sheath gas temperature 400 °C, sheath gas flow 12 l/min and capillary voltage 3500 V. Detection was performed in the multiple reaction monitoring mode (*m/z*, collision energy in parentheses, quantifier underlined): psilocine-d_10_: 215®66 (12), psilocine 205®58 (12); 205®160 (16). Five calibration standards were prepared from human serum with psilocine reference substance (LGC Standards GmbH, Wesel, Germany) and analyzed with the samples. The calibration was linear (regression coefficient >0.999) in the range 1 – 100 ng/ml with limits of detection and quantification below 0.5 ng/ml.

**Preprocessing.**

Data was processed and analysed using the CONN toolbox 18.b[^5^](#_ENREF_5) (<http://www.nitrc.org/projects/conn)> based on SPM12 (<http://www.fil.ion.ucl.ac.uk/spm/)> running in MATLAB 2019a. Before quality assessment, FMRI data was available for 26 subjects in the psilocybin group and 27 subjects in the placebo group.

All functional volumes were realigned, unwarped, segmented into grey and white matter and cerebrospinal fluid, normalised into a standard stereotactic space (Montreal Neurological Institute; MNI) and smoothed with a 6 mm full width at half maximum Gaussian kernel. The first two volumes were excluded in order to to ensure magnetization equilibrium. No slice-time correction was performed as data was acquired using multi-band acquisition.

All individual T_1_-weighted structural volumes were segmented into grey and white matter and cerebrospinal fluid and normalised to MNI space. Noise correction of the functional images included scrubbing with a global signal threshold of z>3 and a composite subject motion threshold of >0.5 mm using ART as implemented in CONN, linear detrending, linear regression of the six motion parameters, and the white matter and cerebrospinal fluid signals, using the individual tissue masks obtained from the T_1_-weighted structural images. Five principal components were extracted from white matter and cerebrospinal fluid signals (using individual tissue masks obtained from the T1-weighted structural images) and removed using CompCor[^6^](#_ENREF_6). These components are thought to reflect noise (especially motion and physiological fluctuations) and are therefore removed from the time series. The resulting functional images were band-pass filtered (0.008 < f < 0.09 Hz) as it was found that band-pass filtering improves independent component results in addition to high-pass filtering (Pignat et al., 2013).

Quality assessment comprised three stages: Firstly, all scans were assessed with regard to the percentage of the scrubbed volumes. Subjects were only included in further analysis, if ≥ 5 min of the scan remained after scrubbing (corresponding to < 83.6% of the initial volumes). This was based on literature indicating that resting state scans < 5 min are not reliable (Birn et al., 2013). Secondly, head motion after scrubbing was assessed using maximum framewise displacement (FD; sphere radius 50 mm) calculated according to Power et al. (Power et al., 2012). Subjects were excluded if maximum FD was > 0.75 mm (half-voxel size). Four subjects (psilocybin group: 3; placebo group: 1) were excluded based on the first criterium and one subject (psilocybin group) was excluded based on the second criterium. The final sample thus consisted of 22 subjects in the psilocybin group and 26 subjects in the placebo group. All further analyses were based on this sample. Furthermore, we tested for significant differences between the groups of this sample. Mean FD before scrubbing was 0.18 mm (± SD 0.05) in the psilocybin group and 0.18 mm (± SD 0.06) in the placebo group. Average maximum FD before scrubbing in the psilocybin group was 0.98 mm (± SD 0.78) in the psilocybin group and 0.99 mm (± SD 0.95) in the placebo group. On average 10.90 volumes per scan (± SD 9.49) were scrubbed in the psilocybin group and 10.50 volumes per scan (± SD 9.80) in the placebo group. Mean FD after scrubbing was 0.17 mm (± SD 0.05) in the psilocybin group and 0.17 mm (± SD 0.05) in the placebo group. Average maximum FD before scrubbing in the psilocybin group was 0.51 mm (± SD 0.12) and 0.50 mm (± SD 0.13) in the placebo group. For comparison between groups, Mann-Whitney U tests were performed because the assumption for normality distribution was not met in several cases (assessed with Shapiro-Wilk tests). There were no significant differences between groups for any of these measures (invalid volumes: p=0.77; mean FD before scrubbing: p=0.72; average maximum FD before scrubbing: p=0.81; mean FD after scrubbing: p=0.81; average maximum FD after scrubbing: p=0.70).

**Independent component analysis**. Independent component analysis (ICA) was performed using group-ICA procedures implemented in the CONN toolbox following methods described by Calhoun et at. (Calhoun et al., 2001). ICA results are determined by the chosen number of dimensions, i.e. a higher number of dimensions might result in a higher number of distinct resting state networks compared with a lower number of dimensions. Dimensionality reduction on the subject-level was set to 64. Independent components were restricted to 20 in order to allow comparisons with 10 established resting state network described by Smith et al. (Smith et al., 2009) and previous studies on psilocybin (Carhart-Harris et al., 2013) and LSD (Carhart-Harris et al., 2016; Müller et al., 2018), which also applied comparable restrictions. Decisions regarding the labelling of the networks identified in this data set were based on visual inspection (Kelly  Jr. et al., 2010) and cross-correlation of the unthresholded ICA components with the unthresholded resting state networks described by Smith et al. (<https://www.fmrib.ox.ac.uk/datasets/brainmap+rsns/>).

**Results**

**AUT.** Independent samples t-tests revealed an acute significant decrease in flexibility (psilocybin: -3.74 ± 0.82; placebo: -1.05 ± 0.81; t(56)= -2.23, *p*=.02) elaboration (psilocybin: -0.64 ± 0.42; placebo: 0.34 ± 1.48; t(56)= -1.96, p=.05), ratings of remoteness (psilocybin: -.22 ± 0.05; placebo: .002 ± 0.05; t(56)= -3.09, *p*=.003), clever (psilocybin: -0.05 ± 0.05; placebo: 0.16 ± 0.04; t(56)= -3.36, *p*=.001), and creativity (psilocybin: -0.09 ± 0.04; placebo: 0.13 ± 0.05; t(56)= -3.78, *p*<.001) after psilocybin compared to placebo. At the follow-up, there was a trending significant increase of flexibility (psilocybin: -1.19 ± 0.87; placebo: -3.24 ± 0.61; t(56)= -1.93,*p*=.06), and no change in the other variables (all *p*>.4).

**Post-hoc correlation.** A spearman’s correlation was conducted post-hoc to investigate the relationship between acute feelings of insight, and long-term changes in new ideas generated on the AUT (novel). A significant positive correlation was found r_s_=.296, *p*=.026.

**References**

1 Mason, N. L. *et al.* Me, myself, bye: regional alterations in glutamate and the experience of ego dissolution with psilocybin. *Neuropsychopharmacology*, doi:10.1038/s41386-020-0718-8 (2020).

2 Silvia, P. J. *et al.* Assessing creativity with divergent thinking tasks: Exploring the reliability and validity of new subjective scoring methods. *Psychology of Aesthetics, Creativity, and the Arts* **2**, 68 (2008).

3 Studerus, E., Gamma, A. & Vollenweider, F. X. Psychometric evaluation of the altered states of consciousness rating scale (OAV). *PLoS ONE* **5**, e12412 (2010).

4 Martin, R., Schurenkamp, J., Pfeiffer, H. & Kohler, H. A validated method for quantitation of psilocin in plasma by LC-MS/MS and study of stability. *International journal of legal medicine* **126**, 845-849, doi:10.1007/s00414-011-0652-8 (2012).

5 Whitfield-Gabrieli, S. & Nieto-Castanon, A. Conn: a functional connectivity toolbox for correlated and anticorrelated brain networks. *Brain connectivity* **2**, 125-141, doi:10.1089/brain.2012.0073 (2012).

6 Behzadi, Y., Restom, K., Liau, J. & Liu, T. T. A component based noise correction method (CompCor) for BOLD and perfusion based fMRI. *NeuroImage* **37**, 90-101, doi:10.1016/j.neuroimage.2007.04.042 (2007).
